# Species Identification of *Dracaena* Using the Complete Chloroplast Genome as a Super-Barcode

**DOI:** 10.3389/fphar.2019.01441

**Published:** 2019-11-29

**Authors:** Zhonglian Zhang, Yue Zhang, Meifang Song, Yanhong Guan, Xiaojun Ma

**Affiliations:** ^1^Institute of Medicinal Plant Development, Chinese Academy of Medical Sciences & Peking Union Medical College, Beijing, China; ^2^Yunnan Branch of Institute of Medicinal Plant Development, Chinese Academy of Medical Sciences & Peking Union Medical College, Jinghong, China

**Keywords:** *Dracaena* Vand. ex L., chloroplast genome, identification, super-barcode, Liliaceae

## Abstract

The taxonomy and nomenclature of *Dracaena* plants are much disputed, particularly for several *Dracaena* species in Asia. However, neither morphological features nor common DNA regions are ideal for identification of *Dracaena* spp. Meanwhile, although multiple *Dracaena* spp. are sources of the rare traditional medicine dragon’s blood, the Pharmacopoeia of the People’s Republic of China has defined *Dracaena cochinchinensis* as the only source plant. The inaccurate identification of *Dracaena* spp. will inevitably affect the clinical efficacy of dragon’s blood. It is therefore important to find a better method to distinguish these species. Here, we report the complete chloroplast (CP) genomes of six *Dracaena* spp., *D. cochinchinensis*, *D. cambodiana*, *D. angustifolia*, *D. terniflora*, *D. hokouensis*, and *D. elliptica*, obtained through high-throughput Illumina sequencing. These CP genomes exhibited typical circular tetramerous structure, and their sizes ranged from 155,055 (*D. elliptica*) to 155,449 bp (*D. cochinchinensis*). The GC content of each CP genome was 37.5%. Furthermore, each CP genome contained 130 genes, including 84 protein-coding genes, 38 tRNA genes, and 8 rRNA genes. There were no potential coding or non-coding regions to distinguish these six species, but the maximum likelihood tree of the six *Dracaena* spp. and other related species revealed that the whole CP genome can be used as a super-barcode to identify these *Dracaena* spp. This study provides not only invaluable data for species identification and safe medical application of *Dracaena* but also an important reference and foundation for species identification and phylogeny of Liliaceae plants.

## Introduction

*Dracaena* Vand. ex L., belonging to the Dracaeneae tribe of Liliaceae, comprises approximately 50 species that are mainly distributed in tropical Africa, Australia, and Asia (Chen and Turland, 2000). In China, there are six species of *Dracaena*, mainly distributed in Yunnan, Guangxi, and Hainan Provinces (Chen and Turland, 2000). At present, there are many disputes over the taxonomy of the *Dracaena* plant, especially with regard to several *Dracaena* spp. in Asia that has been studied very little for several decades (Chen and Turland, 2000; Paul et al, 2012), consequently there are many taxononomic and nomenclatural problems and large numbers of unidentified specimens in herbaria (Chen and Turland, 2000; Yan, 2005). In addition, the traditional classification of the genus *Dracaena* is mainly based on leaf sheath, leaf blade central costa, tepals, and flaments (Wilkin et al.,2013). However, the diversity of natural environment in Southeast Asia area results in significant morphological variations of the *Dracaena* species, making it difficult to accurately identify the *Dracaena* species according to the above identification characteristics. As for the molecular identification, previous studies have demonstrated that barcoding sequences (e.g., ITS, *trnL*, *matK*, *psbA-trnH*, and *rbcL*) are also not ideal for accurate identification of *Dracaena* spp. (Li, 2012; Zheng, 2013; Wang et al., 2015).

In application, *Dracaena* species are usually used as ornamental horticultural plants or as source plants of rare medicinal material dragon’s blood (Adolt and Pavlis, 2004; Aslam et al., 2013). Dragon’s blood is a deep red resin that is widely used throughout the world; it is known to enhance immune function, promote skin repair, stop bleeding, and enhance blood circulation (Fan et al., 2014), and it has been utilized as a traditional medicine for wounds, fractures, piles, leucorrhea, diarrhea, stomach and intestinal ulcers, and even some types of cancer in the histories of many cultures (Gupta et al., 2007; Tapondjou et al., 2008; Kougan et al., 2010; Xu et al., 2010). Modern chemical and pharmacological studies have indicated that the flavonoids, stilbene, saponins, and terpenes in dragon’s blood are the main effective compounds (Hu et al., 2001; González et al., 2004; Chen et al., 2012; Mei et al., 2013; Fan et al., 2014). Among the *Dracaena* plant, multiple *Dracaena* species are the source plants of dragon’s blood (Gupta et al., 2007; Lyons, 1974) such as *D. cinnabari* Balf.f, *D. draco* L, *Dracaena cochinchinensis* (Lour.) S.C.Chen and *D. cambodiana* Pierre ex Gagnp (Gupta et al., 2007; Pearson and Prendergast, 2001), but the Pharmacopoeia of the People’s Republic of China has defined *D. cochinchinensis* as the only source plant. At present, dragon’s blood resources in China mainly depend on imported supplies from Thailand, Laos, Myanmar, and other countries. However, the *Dracaena* species in these areas are more abundant, the inaccurate identification of the *Dracaena* species will inevitably affect the clinical application effect of dragon’s blood. Therefore, the identification of molecular markers that can unambiguously distinguish the *Dracaena* spp. is critical for ensuring the beneficial effects of medicinal products.

The chloroplast (CP), an organelle that converts solar energy to carbohydrates through photosynthesis, has important roles in the biosynthesis of starch, fatty acids, amino acids, and pigments (Jansen and Ruhlman, 2012; Zhao et al., 2015; Daniell et al., 2016). The CP genome is a circular DNA molecule that includes a small single-copy (SSC) region, a large single-copy (LSC) region, and two inverted repeats (IRa and IRb) (Sato et al., 1999). It is highly conserved (Tonti-Filippini et al., 2017) in plants and therefore ideal for ecological, evolutionary, and diversity studies (Wicke et al., 2011). Recently, researchers have used the CP genome as a super-barcode to distinguish species (Xia et al., 2016; Chen et al., 2018b), or have screened sequences from the whole CP genome for species identification (Hu et al., 2016; Thode and Lohmann, 2019; Zhang et al., 2017a). At present, several CP genomes from Liliaceae have previously been reported and deposited in the National Center for Biotechnology Information (NCBI) database. However, from the largest genus of tribe Dracaeneae, *Dracaena*, only one CP genome (*D. cambodiana*) has been published previously (Zhu et al., 2018). In this study, we report the CP genomes of six *Dracaena* spp., including *D. cochinchinensis* (Lour.) S.C.Chen, *D. cambodiana* Pierre ex Gagnep, *D. angustifolia* (Medik.) Roxb, *D. terniflora* Roxb., *D. hokouensis* G.Z.Ye, and *D. elliptica* Thunb. & Dalm., obtained *via* high-throughput Illumina sequencing technology. Our aim is to utilize the CP genome as a super-barcode for the identification of *Dracaena* spp. and to provide invaluable genetic information for understanding their phylogenetic relationships and other future studies.

## Materials and Methods

### Plant Materials, DNA Extraction, and Illumina Sequencing

Plant materials from six *Dracaena* spp. were collected from the Yunnan Branch of the Institute of Medicinal Plant Development, Chinese Academy of Medical Sciences; the voucher specimens were deposited in the Institute of Medicinal Plant Development herbarium. Total genomic DNA was extracted from clean leaves from samples frozen at −80°C using the TaKaRa MiniBEST Universal Genomic DNA Extraction Kit (TaKaRa, Kusatsu, Japan) with a standard protocol, DNA quality was assessed using a Nanodrop 2000 (Thermo Scientific, Waltham, MA, USA) and by electrophoresis in a 1% (w/v) agarose gel. The OD_260/280_ values ranged from 1.8 to 2.0, and >2 µg of DNA was equally pooled from individuals of the six species to generate shotgun libraries. cpDNA samples were randomly sheared, incubated with fragmentation buffer, and broken into 300–500-bp fragments in an M220 focused ultrasonicator (Covaris, Woburn, MA, USA). Connect the A & B connector at both ends of the DNA fragment, Screen the segments, then remove the self-connecting segments of the connector. Fragment screening by electrophoresis in agarose gel, keep the fragment with A connector at one end and B connector at the other end, which subsequent addition of NaOH to denatures, produce single-stranded DNA fragments. The library was sequenced using Illumina HiSeq 4000 sequencing platform at the Major Company (Shanghai, China).

### Genome Assembly and Annotation

Raw data for the six *Dracaena* spp. were generated with a paired-end read length of 150 bp. The low-quality reads (Q < 20) were filtered out and the clean reads were used for CP genome assembly. A reference database was created by downloading all plant CP sequences from NCBI. The high-quality reads were mapped to the database, and the mapped reads were extracted based on sequence coverage and similarity. The extracted reads were assembled into contigs using SOAPdenovo2 (Luo et al., 2012), and the resulting contigs were combined and extended to obtain complete CP genome sequence. Finally, the positions of the LSC, SSC, and two IR regions of the CP genomes were determined by localization, and complete CP genomes were obtained. Annotation of the six complete *Dracaena* CP genomes were executed using the online program Dual Organellar GenoMe Annotator (DOGMA, http://dogma.ccbb.utexas.edu/) (Wyman et al., 2004) coupled with manual correction. The tRNAscan-SE software (v2.0, University of California, Santa Cruz, CA, USA) (Schattner et al., 2005) and DOGMA (Wyman et al., 2004) were used to identify the tRNA genes. The BLAST versus reference sequences was used for verifying boundaries genes, intron/exon and coding regions. Moreover, MEGA 6.0 was used to analyze the GC content (Tamura et al., 2013). The Organellar Genome DRAW (OGDRAW) (v1.2, Max Planck Institute of Molecular Plant Physiology, Potsdam, Germany) (Lohse et al., 2007) was used with default settings to draw the gene map, which was then checked manually. Finally, a.sqn file was generated to submit the sequences to NCBI. The complete and correct CP genome sequences of the six *Dracaena* species were deposited in GenBank, accession numbers are MN200193 (*D. angustifolia*), MN200194 (*D. cambodiana*), MN200195 (*D. cochinchinensis*), MN200196 (*D. elliptica*), MN200197 (*D. hokouensis*), and MN200198 (*D. terniflora*) respectively.

### Genome Structure and Comparative Genome Analysis

The CodonW software (University of Texas, Houston, TX, USA) was used to investigate the distribution of codons based on the relative synonymous codon usage (RSCU) ratio (Sharp and Li, 1987). Conserved sequences were identified between the CP genomes of *D. cochinchinensis* and those of *D. cambodiana*, *D. angustifolia*, *D. terniflora*, *D. hokouensis*, and *D. elliptica* by BLASTN analysis with an E-value cutoff of 1e−10. The mVISTA (Frazer et al., 2004) program was used in Shuffle-LAGAN mode to compare the six *Dracaena* CP genomes using the *D. cochinchinensis* CP genome as a reference.

### Repeat Sequence Analysis

RSCU value, which is the ratio between the frequency of use and the expected frequency of a particular codon, was used to detected non-uniform synonymous codon usage within a coding sequence (Sharp and Li, 1987). Moreover, simple sequence repeats (SSRs) were detected using the MISA software (http://pgrc.ipk-gatersleben.de/misa/), with parameters set to encompass the number of repeat units of a mononucleotide SSR ≥10 nucleotides in length, followed by ≥5 and ≥4 repeat units for di- and tri-nucleotide SSRs, respectively, and ≥3 repeat units for tetra-, penta-, and hexa-nucleotide SSRs. The sizes and locations of repeat sequences in the CP genomes of the six *Dracaena* spp. were identified using REPuter (University of Bielefeld, Bielefeld, Germany) (Kurtz et al., 2001) with the parameters set to a similarity percentage of scattered repeat copies ≥90% and a minimal repeat size of 30 bp.

### Phylogenetic Analysis

To determine phylogenetic positions of the six *Dracaena* species within Liliaceae, we analyzed the CP genomes of 37 species, encompassing 31 additional taxa within this lineage. The CP genome sequences of 31 species were downloaded from NCBI. The sequences were initially compared using MAFFT (Katoh et al., 2005), followed by multiple sequence visual analysis and manual adjustment using BioEdit (Hall, 1999). We used the CP genomes of *Stemona japonica* (Blume) Miq. (NC_039675.1) as outgroups and constructed phylogenetic trees employing 37 CP genomes sequences using the maximum parsimony (MP) and neighbor-joining (NJ) methods in MEGA 6.0 (Tamura et al., 2013) with 1000 bootstrap replicates.

## Results and Discussion

### CP Genomes of Six *Dracaena* spp.

**Figure 1:**
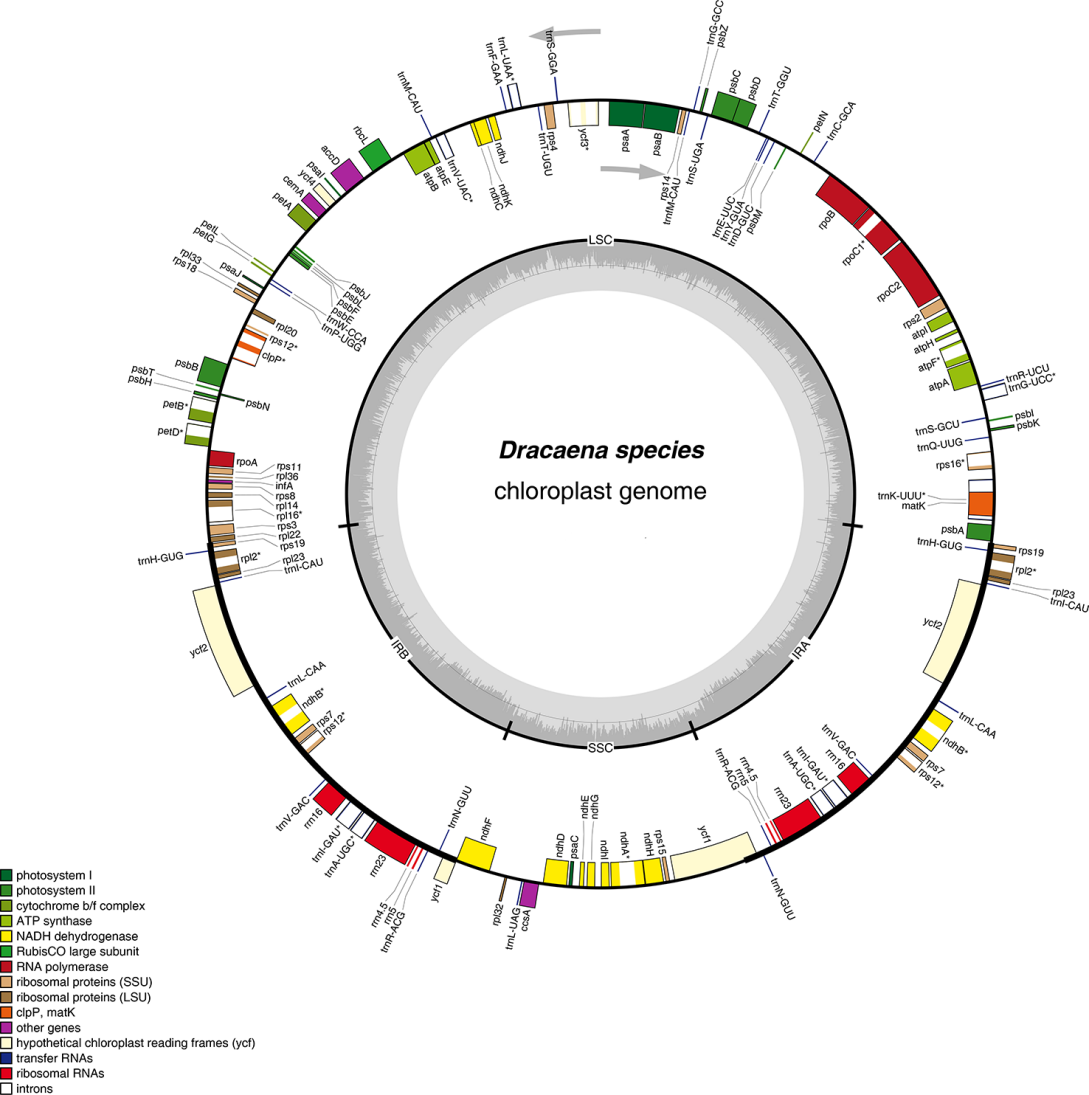
Gene map of the complete chloroplast genomes of *Dracaena* species. Genes on the inside of the circle are transcribed clockwise, and those on the outside are transcribed counter-clockwise. The darker gray area in the inner circle corresponds to GC content, whereas the lighter gray corresponds to AT content.

At present, complete CP genome sequencing is feasible with the genome skimming approach as a result of the wide application of next-generation DNA sequencing technology and bioinformatics software tools (Twyford and Ness, 2016). Illumina technology can be used for assembling the complete CP genome without the tedious of separating CP DNA from nuclear DNAs (Wang et al., 2016a). The raw data from the six *Dracaena* species is 7.04 Gb for *D. cochinchinensis*, 7.02 Gb for *D. cambodiana*, 6.59 Gb for *D. angustifolia*, 6.24 Gb for *D. terniflora*, 7.74 Gb for *D. hokouensis*, and 6.88 Gb for *D. elliptica*. The genome sequences assembled using the reads obtained from the Illumina sequencing platform ranged from 155,055 bp for *D. elliptica* to 155,449 bp for *D. cochinchinensis* (**Table 1**), which are similar to other Liliaceae CP genomes (Kim et al., 2016; Park et al., 2017; Liu et al., 2018). The genome exhibited typical cyclic tetramer structure, including the SSC and LSC regions separated by two IR regions (**Figure 1**), and the four regions from the six species had similar lengths (**Table S1**). The SSC lengths ranged from 18,456 to 18,494 bp, which is larger than those from the six *Ipomoea* species (Park et al., 2018) and *Strobilanthes cusia* (Nees) Kuntze (Chen et al., 2018a). Meanwhile, the LSC lengths ranged from 83,621 to 83,907 bp, which is shorter than those from the six *Ipomoea* species and *S. cusia*. In addition, the IR lengths each ranged from 26,489 bp to 26,530 bp, which is larger than those obtained from *S. cusia* (Chen et al., 2018a), *Taxillus chinensis* (DC.) Danser (Li et al., 2017), and *T. sutchuenensis* (Lecomte) Danser (Li et al., 2017). Moreover, the GC contents of the six *Dracaena* CP genomes were similar, and the IR regions (42.9%) had higher GC contents than the single-copy regions (LSC: 35.4%–35.6% and SSC: 31.1%–31.2%). This result is consistent with previous reports (Park et al., 2018; Li and Zheng, 2018). The high GC contents in the IR regions may be attributable to the presence of four rRNA genes (*rrn16*, *rrn23*, *rrn4*.5, and *rrn5*) (Chen et al., 2018b).

**Table 1:** Summary statistics for assembly of the six complete chloroplast (CP) genomes of *Dracaena* species.

Species names	*D. cochinchinensis*	*D. cambodiana*	*D. angustifolia*	*D. terniflora*	*D. hokouensis*	*D. elliptica*
Raw reads	50,369,222	50,223,744	47,183,736	44,650,560	55,383,360	49,214,248
raw data (bp)	7,555,383,300	7,533,561,600	7,077,560,400	6,697,584,000	8,307,504,000	7,382,137,200
Mapped CP reads	2,818,312	1,539,342	1,561,310	3,249,348	2,238,882	3,657,230
Size (bp)	155,449	155,291	155,332	155,347	155,340	155,055
LSC length (bp)	83,907	83,752	83,807	83,794	83,796	83,621
SSC length (bp)	18,492	18,489	18,465	18,493	18,494	18,456
IR length (bp)	53,050	53,050	53,060	53,060	53,050	52,978
Coding (bp)	77,187	77,202	78,732	78,744	78,744	77,130
Non-coding (bp)	78,262	78,089	76,600	76,603	76,596	77,925

CP, complete chloroplast; IR, inverted repeat; LSC, large single-copy; SSC, small single-copy.

Furthermore, the six *Dracaena* CP genomes have similar gene contents, sequences, and orientations, which are the typical characteristics of higher plant CP genomes (Wicke et al., 2011; Qian et al., 2013). The genes in the six CP genomes consisted of 84 protein-coding genes, 38 tRNA genes, and 8 rRNA genes. Among them, seven protein-coding genes (*rps19*, *rpl2*, *rpl23*, *ycf2*, *ndhB*, *rps7*, and *rps12*), four rRNA genes (*rrn4*.5×2, *rrn5*×2, *rrn16*×2, and *rrn23*×2), and eight tRNA genes (*trnR*-*ACG*, *trnA*-*UGC*, *trnI*-*GAU*, *trnV*-*GAC*, *trnL*-*CAA*, *trnI*-*CAU*, *trnH*-*GUG*, and *trnN*-*GUU*), as well as the IR regions, contained two repeat units, similar to *Ligularia intermedia* Nakai (Chen et al., 2018b) and *Artemisia annua* L. (Shen et al., 2017). Moreover, 18 of the genes contained introns, and among them, 10 protein-coding genes (*petB*, *petD*, *atpF*, *ndhA*, *ndhB*, *rpoC1*, *rps12*, *rps16*, *rpl16*, and *rpl2*) and 6 tRNA genes contained a single intron, and 2 protein-coding genes (*clpP* and *ycf3*) contained two introns (**Table 2**). Introns play an important role in the regulation of gene expression by enhancing the expression of exogenous genes at specific sites and at specific times in plants (Xu et al., 2003). In total, the coding regions including protein-coding genes, tRNAs, and rRNAs occupied 57.35%–58.37% of the six *Dracaena* CP genomes, while the non-coding regions constituted 41.63%–42.68% of the genomes.

**Table 2:** List of genes found in the six CP genomes of *Dracaena* species.

No.	Group of genes	Gene names	Number of genes
1	Photosystem I	psaA, *psaB*, *psaC*, *psaI*, *psaJ*	5
2	Photosystem II	psbA, *psbB*, *psbC*, *psbD*, *psbE*, *psbF*, *psbH*, *psbI*, *psbJ*, *psbK*, *psbL*, *psbM*, *psbN*, *psbT*, *psbZ*	15
3	Cytochrome b/f complex	petA, petB*, petD*, *petG*, *petL*, *petN*	6
4	ATP synthase	atpA, *atpB*, *atpE*, atpF*, *atpH*, *atpI*	6
5	NADH dehydrogenase	ndhA*, ndhB*(×2), *ndhC*, *ndhD*, *ndhE*, *ndhF*, *ndhG*, *ndhH*, *ndhI*, *ndhJ*, *ndhK*	11
6	RubisCO large subunit	rbcL	1
7	RNA polymerase	rpoA, *rpoB*, rpoC1*, *rpoC2*	4
8	Ribosomal proteins (SSU)	rps2, *rps3*, *rps4*, *rps7*(×2), *rps8*, *rps11*, rps12*(×2), *rps14*, *rps15*, rps16*, *rps18*, *rps19*(×2)	12
9	Ribosomal proteins (LSU)	rpl2*(×2), *rpl14*, rpl16*, *rpl20*, *rpl22*, *rpl23*(×2), *rpl32*, *rpl33*, *rpl36*	9
10	Other genes	accD, clpP**, *matK*, *ccsA*, *cemA*	5
11	Proteins of unknown function	ycf1, *ycf2*(×2), ycf3**, *ycf4*	4
12	Transfer RNAs	38 *tRNA*s (6 contain an intron, 8 in the IRs)	
13	Ribosomal RNAs	rrn4.5(×2), *rrn5*(×2), *rrn16*(×2), *rrn23*(×2)	

*Gene contains one intron. **Gene contains two introns. (×2) indicates the number of the repeat unit is 2.

### Codon Usage

RSCU values reveal biases in synonymous codon usage. The codon usage and anti-codon recognition patterns of the six *Dracaena* CP genomes are shown in **Table S2**. The CP protein-coding genes of these six species contained 61 codons encoding 20 amino acids. The coding regions (CDS) comprised 25,710 codons in *D. elliptica* to 26,248 codons in *D. terniflora* and *D. hokouensis*. Codons for isoleucine (5.63%∼5.89%) and lysine (5.41%∼5.66%) were the most abundant in the six *Dracaena* CP genomes, whereas those for cysteine (0.37∼∼ 0.39%) and arginine (0.44∼∼0.48%) were observed least often, at lower frequencies than in artichoke (Curci et al., 2015) and the six *Ligularia* species (Chen et al., 2018b).

An RSCU value <1.00 indicates that the codon usage frequency is lower than expected, whereas an RSCU value >1.00 indicates that the codon usage frequency is higher than expected (Sharp and Li, 1987). In this study, other than leucine and isoleucine, amino acid codons in the CP genomes of the six *Dracaena* spp. preferentially ended with A or U (RSCU >1). Codons ending in A and/or U accounted for 69.76% (*D. cochinchinensis*) to 72.87% (*D. terniflora*) of all CDS codons in the six CP genomes. These results are similar to those observed for *Papaver rhoeas* L.and *Papaver orientale* L. (Zhou et al., 2018). The codon usage pattern may be determined by the high proportion of A/T composition bias. In other terrestrial higher plant CP genomes, high codon preference in codon usage, especially A/T bias, is very common (Kim and Lee, 2004; Qian et al., 2013). This phenomenon indicates that stable CP evolution is beneficial for protecting important CP genes from harmful mutations and adaptation to selection stress (Wang et al., 2016b; Ivanova et al., 2017; Zuo et al., 2017).

Meanwhile, codons ending in A and/or T (U) usually had high RSCU values in the six CP genomes, e.g., GCU (1.79) for alanine, UCU (1.69) for serine, and GGA (1.64) for glycine. In addition, the use of the start codon (ATG) and TGG encoding Trp showed no bias in the six CP genomes (RSCU = 1), and the remaining amino acids have preferred codons. Furthermore, protein-coding genes accounted for 49.65%∼50.69% of the whole genome sequence, and tRNA genes accounted for 1.849%∼1.853% (**Table S3**). A total of 18 genes contained introns in the six *Dracaena* CP genomes, including 12 protein-coding genes (*rps16*, *atpF*, *rpoC1*, *ycf3*, *rps12*, *clpP*, *petB*, *petD*, *rpl16*, *rpl2*, *ndhB*, and *ndhA*), and 6 tRNA genes (*trnK-UUU*, *trnG-UCC*, *trnL-UAA*, *trnV-UAC*, *trnl-GAU*, and *trnA-UGC*; **Table S4**) were identified in this research. *TrnK-UUU* has the longest intron (857 bp).

### SSRs in the CP Genomes of the Six *Dracaena* spp.

**Figure 2:**
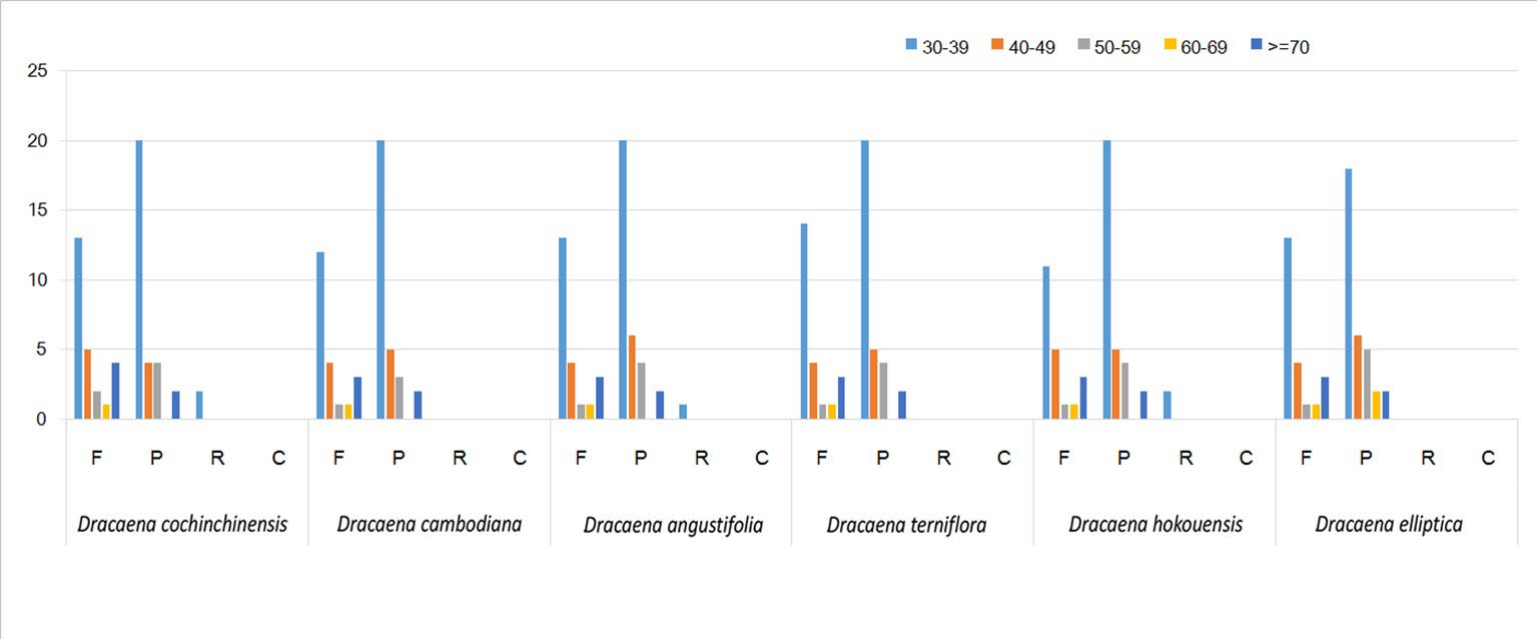
Repeat analysis in six *Dracaena* complete chloroplast (CP) genomes. REPuter was used to identify repeat sequences with length ≥30 bp and sequence identified ≥90% in the CP genomes. F, P, R, and C indicate the repeat types F (forward), P (palindrome), R (reverse), and C (complement), respectively. Repeats with different lengths are indicated in different colors.

SSRs are widely present in CP genomes, consisting of 1–6-nucleotide repeat units, and are valuable molecular markers of high variation within the same species (Powell et al., 1995). The repeats were divided into tandem repeats and dispersed repeats, and the dispersed repeats can be further divided into four repetition types: complement, forward, reverse, and palindromic repeats (Kurtz et al., 2001). In this study, we analyzed repeats using the software tools Tandem Repeats Finder and REPuter, and the distributions of repeated sequences and SSRs in the CP genomes of the six species were analyzed. The repeat structure analysis is shown in **Figure 2**. The results showed that the numbers of repeat types in the six *Dracaena* spp. were extremely similar. Among the repeat types, palindromes (29∼33) were the most abundant, followed by forward (21∼25) and reverse repeats (0∼2), and there were no complement repeats in the six *Dracaena* CP genomes. Furthermore, totals of 69, 69, 67, 64, 70, and 71 SSRs were identified using the microsatellite identification tool (MISA) from the CP genomes of *D. cochinchinensis*, *D. cambodiana*, *D. angustifolia*, *D. terniflora*, *D. hokouensis*, and *D. elliptica*, respectively (**Table 3**).

**Table 3:** The simple sequence repeat (SSR) types of the six CP genomes of *Dracaena* species.

SSR type	Repeat unit	Amount					
		*D. cochinchinensis*	*D. cambodiana*	*D. angustifolia*	*D. terniflora*	*D. hokouensis*	*D. elliptica*
Mono	A/T	36	37	37	34	41	44
Mono	C/G	2	2	1	1	1	0
Di	AG/CT	3	3	3	3	3	3
Di	AT/AT	11	11	12	12	11	11
Tri	AAG/CTT	1	1	1	1	1	1
Tri	AAT/ATT	2	2	2	2	2	2
Tetra	AAAG/CTTT	2	2	2	2	2	2
Tetra	AAAT/ATTT	4	4	4	4	4	4
Tetra	AATC/ATTG	2	2	2	2	2	2
Tetra	AATG/ATTC	1	1	1	1	1	1
penta	AAACG/CGTTT	2	2	0	0	0	0
penta	ACTAT/AGTAT	1	0	0	0	0	0
Hexa	AATTAT/AATTAT	2	2	1	0	1	1
Hexa	AAGATT/AATCTT	0	0	1	1	1	0
Hexa	AAAAGT/ACTTTT	0	0	0	1	0	0

It is well known that the repeat sequences are associated with plastome tissues, mostly in the intergenic and intron regions, and only a small portion is distributed in the genetic region (Salih et al., 2017; Zhou et al., 2017). In the present study, SSRs were less abundant in the CDS (15∼16 SSRs) of the six *Dracaena* CP genomes than in the non-coding region. In addition, the types and distributions of the potential SSRs were investigated. The most abundant type was repeated mononucleotide (54.69%∼61.97%), which were found 14∼15 times in the six *Dracaena* species. These were followed by dinucleotide (19.72%∼23.44%), trinucleotide (4.23%∼4.69%), tetranucleotide (12.68%∼14.06%), pentanucleotide (0%∼4.35%), and hexanucleotide repeats (1.41%∼2.99%). Among these repeats, the A/T repeat was the most abundant motif, followed by AT/TA dinucleotide repeats, then AAAT/ATTT tetranucleotide repeats. These results were consistent with previous reports that CP SSRs usually consist of short poly-A or poly-T repeats and rarely contain tandem G or C repeats in many plants (Kuang et al., 2011). In addition, with the exceptions of *D. cochinchinensis* and *D. cambodiana*, the other four species had no pentanucleotide SSRs (**Table 3**). At present, SSR markers are widely used in genetic diversity and population structure assessments, comparative genomics, development of genetic maps, and marker-assisted selective breeding (Chen et al., 2015; Zhou et al., 2018; Zhang et al., 2017b). The repeats identified in this study will provide valuable resources for species identification and population studies of *Dracaena* spp.

### Comparative Genome Analysis

**Figure 3:**
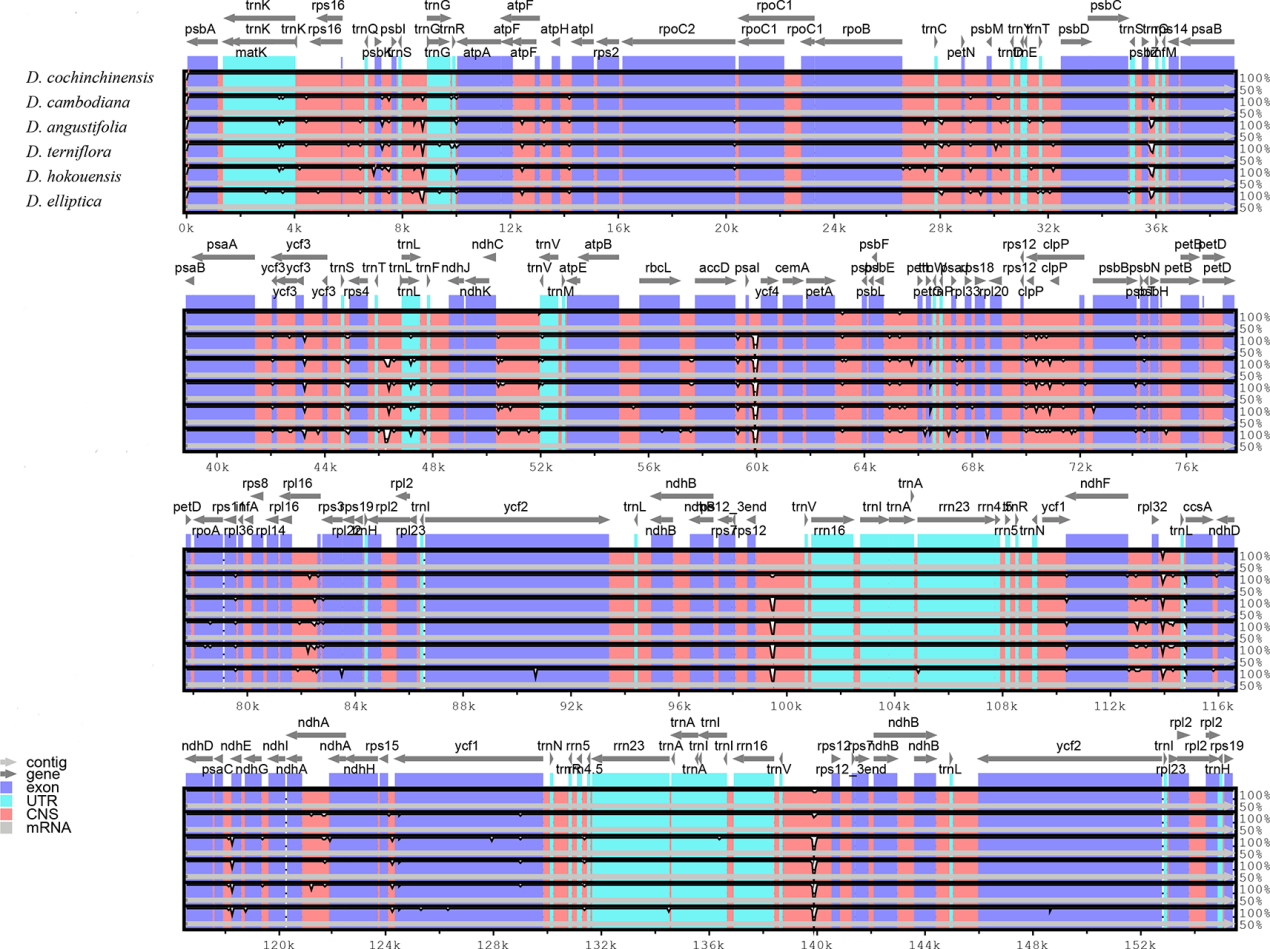
Structure comparison of the six *Dracaena* CP genomes by using the mVISTA program. Gray arrows and thick black lines above the alignment indicate genes with their orientation and the position of the IRs, respectively. A cut-off value of 70% identity was used for the plots, and the Y-scale represents the percent identity between 50% and 100%.

Comparative analysis of CP genome is advantageous for understanding the genetic diversity and evolutionary relationships of plants in different environments (Daniell et al., 2016; Zhao et al., 2018). The sequence homology of the six *Dracaena* CP genomes was compared and analyzed using the mVISTA software, and *D. cochinchinensis* was used as a reference sequence (**Figure 3**). The comparison revealed that some differences between *D. cochinchinensis* and the other five *Dracaena* spp. existed within intergenic spacers (IGS), such as *rps12*-*trnV* and *psaI*-*ycf4*; and the CP genomes of the other five *Dracaena* spp. were not significantly different., only some differences existed in the *ycf1* gene and intergenic regions, such as *trnS-trnG*, *rpoB-trnC*, *rpl16-rps3*, *psbE-petL*, and *ndhF-rpl32*.

As shown in **Table 4**, the gene with the most variable sites in coding regions is *ycf1*, which has 73 variable sites and 35 indels within the 5,466 bp alignment. the intergenic regions with the most variable sites is rps7 → trnV-GAC, which has 59 variable sites and 16 indels within the 2,716 bp length. On the whole, the variable sites of the six *Dracaena* CP genomes are more than some other species (Zhang et al., 2017c). Furthermore, we found that the majority of sequence variations were within the LSC and SSC regions, whereas the IR regions had relatively fewer sequence variations. This result further supports the findings of previous studies: the two IR regions are more conserved than the LSC and SSC regions (Nazareno et al., 2015; Li et al., 2017; Lu et al., 2017). This may be because mutations in the IR sequences are corrected by gene conversion (Khakhlova and Bock, 2006). In addition, the sequence variation in the non-coding regions was higher than in the gene coding regions in the CP genomes of the six *Dracaena* spp., which is consistent with previous findings for most gymnosperms (Zhang et al., 2011; Lu et al., 2017; Zhao et al., 2018).

**Table 4:** The 10 most-divergent coding regions and intergenic regions in the six Dracaena species.

Regions	Genes	Length	Variable sites	Indels	Percentage of identical sites (%)
Coding regions	ycf1	5466	73	35	99.15
	clpP	2052	48	90	97.58
	ccsA	972	45	50	96.05
	ndhD	1521	25	8	99.1
	rbcL	1440	21	14	98.89
	psbB	1527	15	26	99
	psbF	120	12	0	94.88
	rps18	306	8	7	97.62
	rps16	210	5	0	98.98
	psbK	186	4	11	96.86
Intergenic regions	rps7 → trnV-GAC	2716	59	16	98.54
	rps12 → trnV-GAC	1859	57	16	97.9
	trnS-GCU → trnG-UCC	1026	24	90	95.1
	rpl32 → trnL-UAG	873	21	56	97.03
	trnT-UGU → trnL-UAA	932	17	153	95
	trnC-GCA → petN	923	17	30	97.66
	trnT-GGU → psbD	743	17	15	98.24
	psbE → petL	1315	15	36	98.4
	trnF-GAA → ndhJ	723	14	12	98.54
	trnP-UGG → psaJ	362	13	6	97.41

### Identification and Phylogenetic Analysis of *Dracaena* Species

The highly variable regions of CP genomes can be used as potential DNA barcodes for species identification and phylogenetic analyses, such as in *Fritillaria* species (Li et al., 2016). Our results showed high similarity among the six sequences. The largest change in gene length occurred in ndhF, with 2,211 bp in *D. hokouensis*, 2,214 bp in *D. elliptica*, 2,220 bp in *D. cochinchinensis*, 2,223 bp in *D. angustifolia* and *D. terniflora*, and 2,244 bp in *D. cambodiana*. Furthermore, the most divergent regions between the six *Dracaena* species localized in the IGS among the CP genomes, but these variable regions is not enough to distinguish the six *Dracaena* species. As there are no significant difference in structure and size of the six CP genome sequence of *Dracaena* species, we thought that the complete CP genome could be used as a barcode marker to distinguish *Dracaena* species.

**Figure 4:**
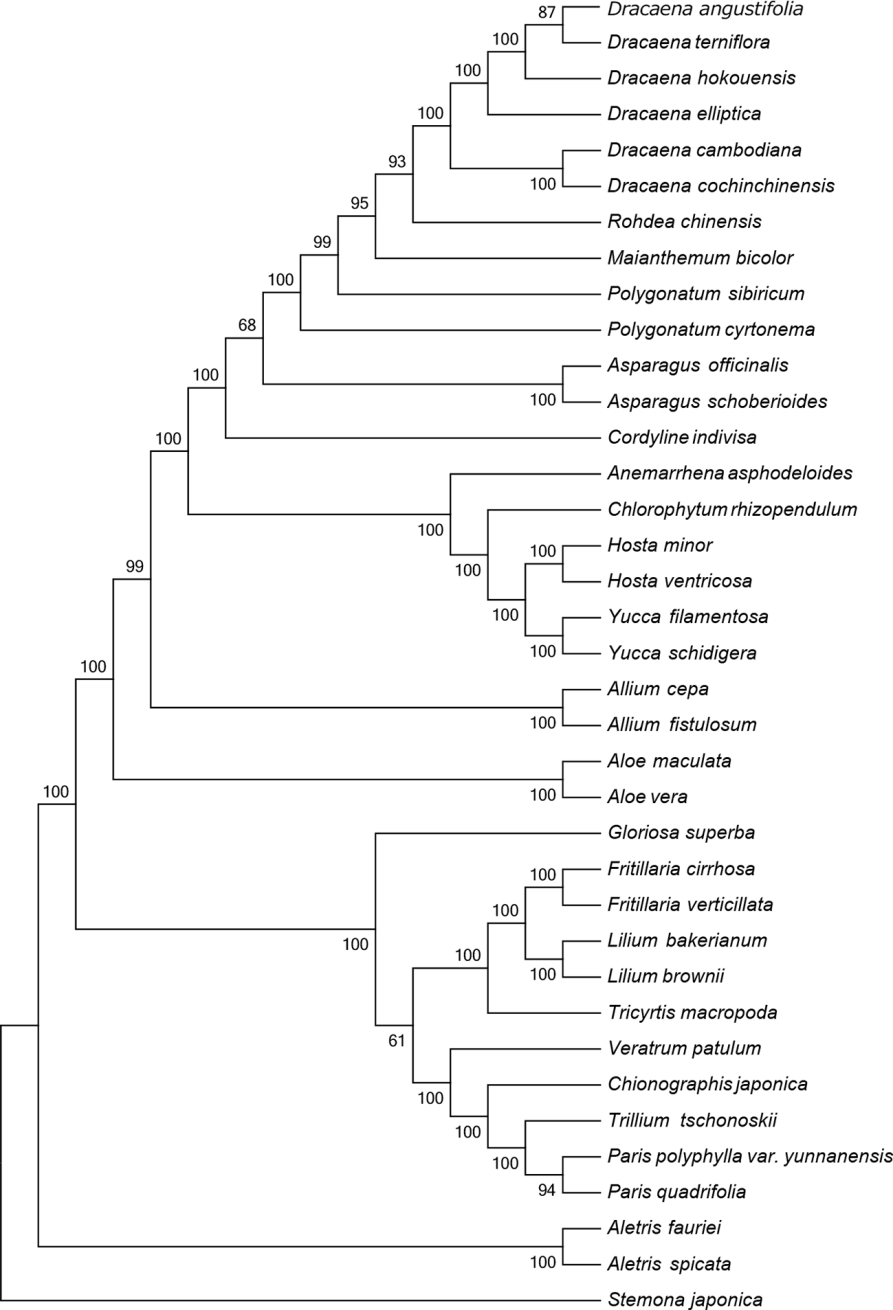
Phylogenetic tree constructed using maximum parsimony (MP) based on complete CP genomes of six *Dracaena* and other 31 species. Numbers above the branches are the bootstrap support values.

The CP sequence is crucial for studying phylogenetic relationships and determining taxonomic status among angiosperms (Jansen et al., 2007). To identify the phylogenetic positions of the six *Dracaena* spp. within the Liliaceae, we obtained 30 complete CP genome sequences belonging to 20 genera of the family Liliaceae (including two species each from *Aletris*, *Allium*, *Aloe*, *Asparagus*, *Hosta*, *Yucca*, *Polygonatum*, *Fritillaria*, *Lilium*, and *Paris*, and one species each from *Anemarrhena*, *Chlorophytum*, *Maianthemum*, *Rohdea*, *Cordyline*, *Tricyrtis*, *Chionographis*, *Trillium*, *Veratrum*, and *Gloriosa*) and one species belonging to Stemonaceae from the RefSeq database (**Table S5**). In the MP (**Figure 4**) and NJ (**Figure S1**) tree, the six species of the genus *Dracaena* are clustered together and separated from the other 20 genera of the family Liliaceae, indicating the close relationship of the six *Dracaena* species. Meanwhile, the six *Dracaena* spp. were distinct from one another, with support values ≥85%; *D. angustifolia* and *D. terniflora* were clustered together, *D. hokouensis* exhibited a sister relationship, and *D. cambodiana* and *D. cochinchinensis* were clustered together. The results showed that the CP genomes can be used to identify the six *Dracaena* species. In addition, we explored the construction of a phylogenetic tree based on 36 complete Liliaceae CP genomes sequence multiple alignments in present study. Our results will provide an important reference and foundation for species identification and phylogeny of Liliaceae plants.

## Conclusion

This study reported the CP genomes from six *Dracana* species, range from 155,055 bp (*D. elliptica*) to 155,449 bp (*D. cochinchinensis*), and the structure and composition of the CP genomes are highly similar. The CP genomes genes consisted of 84 protein-coding genes and 38 tRNAs, with 8 rRNA genes in the six genomes. Among the six *Dracana* species, *D. angustifolia* had the closest relationship with *D. terniflora*, *D. cochinchinensis* had the closest relationship with *D. cambodiana* respectively. The MP and NJ tree showed that the CP genome can be used to identify the six *Dracana* species and is expected to become a super-barcode for the identification of *Dracana* species.

## Data Availability Statement

The datasets generated for this study can be found in GenBank, accession numbers are MN200193 (*D. angustifolia*), MN200194 (*D. cambodiana*), MN200195 (*D. cochinchinensis*), MN200196 (*D. elliptica*), MN200197 (*D. hokouensis*) and MN200198 (*D. terniflora*).

## Author Contributions

ZZ and XM conceived and design the study. XM acquired the funding. ZZ and YG collected samples and determined the species. YZ performed the genome assembly and analysis on the data. ZZ wrote the manuscript. XM supervised the manuscript. All authors have read and approved the final manuscript.

## Funding

This research was funded by CAMS Initiative for Innovative Medicine (No. 2017-12M-B&R-09) and Quality guarantee system of Chinese herbal medicines (No. 201507002).

## Conflict of Interest

The authors declare that the research was conducted in the absence of any commercial or financial relationships that could be construed as a potential conflict of interest.
