# Corrigendum: Species Identification of *Dracaena* Using the Complete Chloroplast Genome as a Super-Barcode

**DOI:** 10.3389/fphar.2020.00051

**Published:** 2020-02-13

**Authors:** Zhonglian Zhang, Yue Zhang, Meifang Song, Yanhong Guan, Xiaojun Ma

**Affiliations:** ^1^Institute of Medicinal Plant Development, Chinese Academy of Medical Sciences & Peking Union Medical College, Beijing, China; ^2^Yunnan Branch of Institute of Medicinal Plant Development, Chinese Academy of Medical Sciences & Peking Union Medical College, Jinghong, China

**Keywords:** *Dracaena* Vand. ex L., chloroplast genome, identification, super-barcode, Liliaceae

In the original article, the figure legends were associated with the wrong figures. **Figure 4** should be **Figure 2**, **Figure 2** should be **Figure 3**, and **Figure 3** should be **Figure 4**. The legends remain the same. The figures and their correct legends appear below.

**Figure 2:**
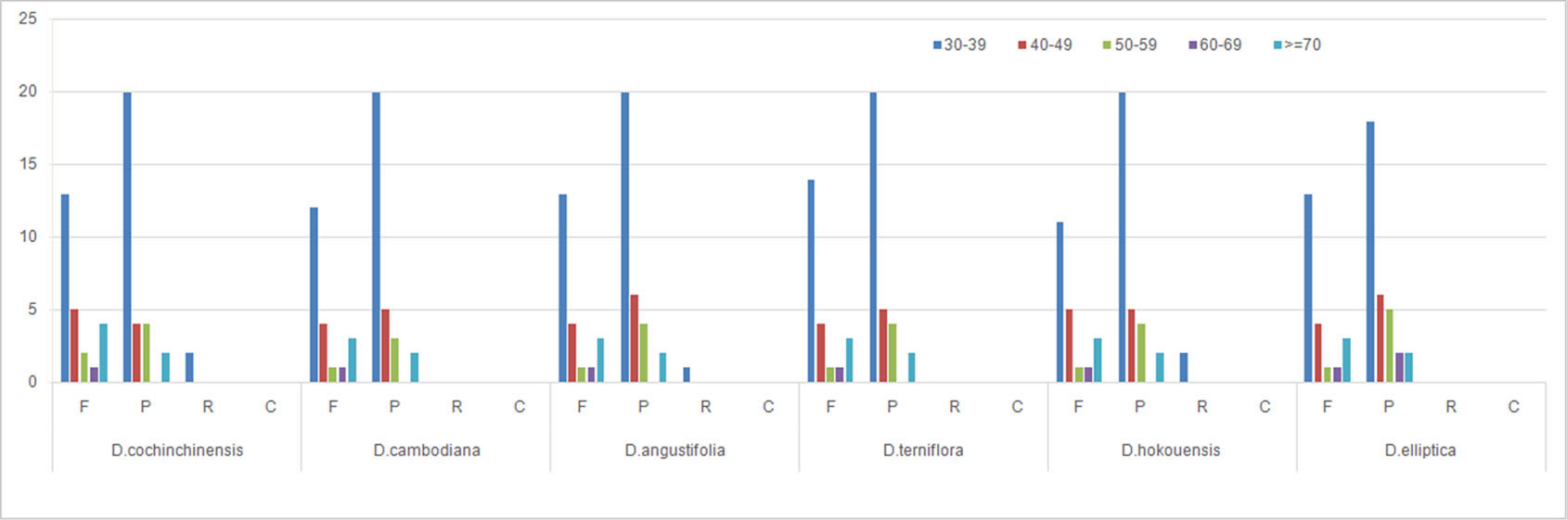
Repeat analysis in six *Dracaena* CP genomes. REPuter was used to identify repeat sequences with length ≥30 bp and sequence identified ≥90% in the CP genomes. F, P, R, and C indicate the repeat types F (forward), P (palindrome), R (reverse), and C (complement), respectively. Repeats with different lengths are indicated in different colors.

**Figure 3:**
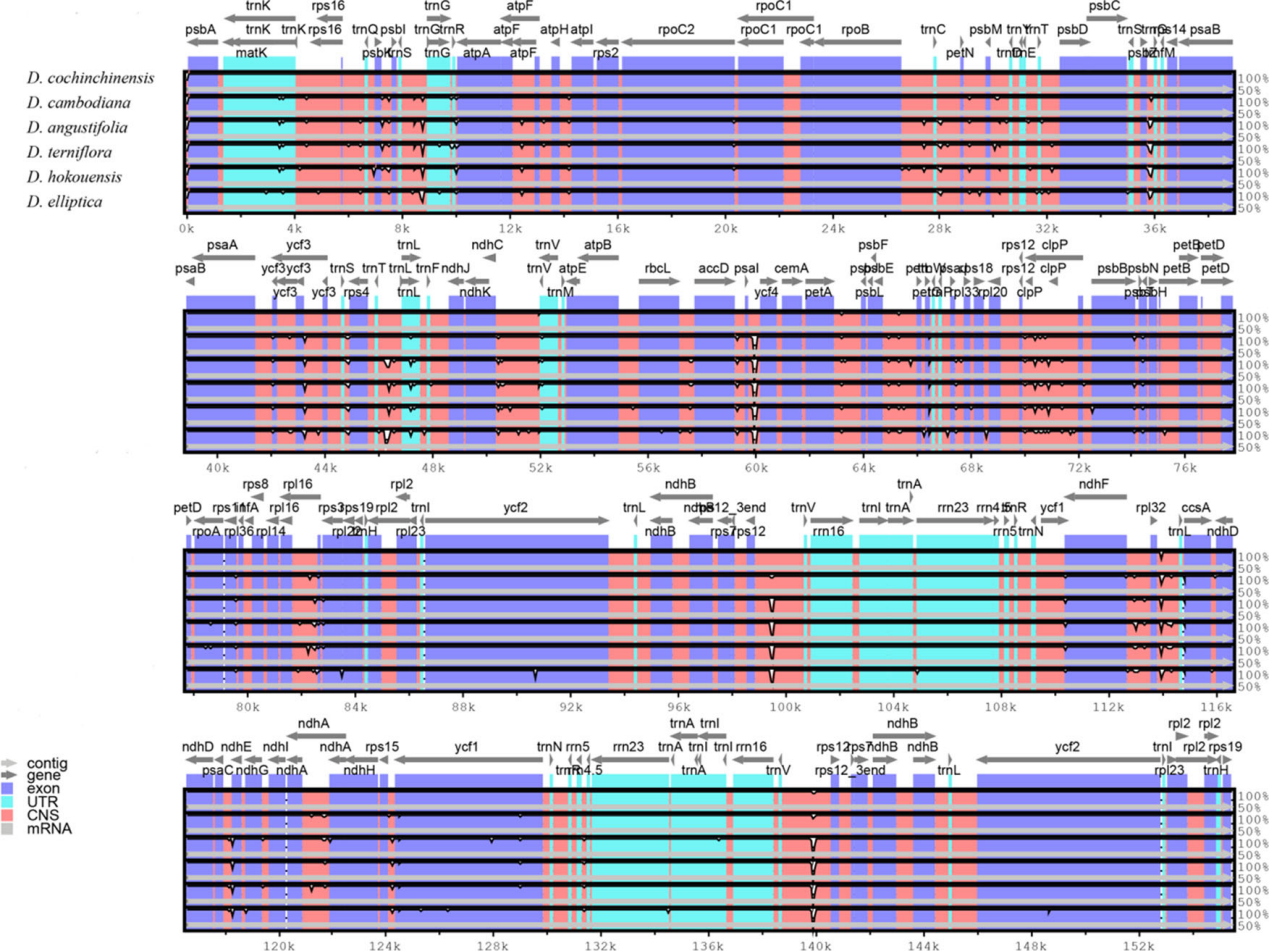
Structure comparison of the six *Dracaena* CP genomes by using the mVISTA program. Gray arrows and thick black lines above the alignment indicate genes with their orientation and the position of the IRs, respectively. A cut-off value of 70% identity was used for the plots, and the Y-scale represents the percent identity between 50% and 100%.

**Figure 4:**
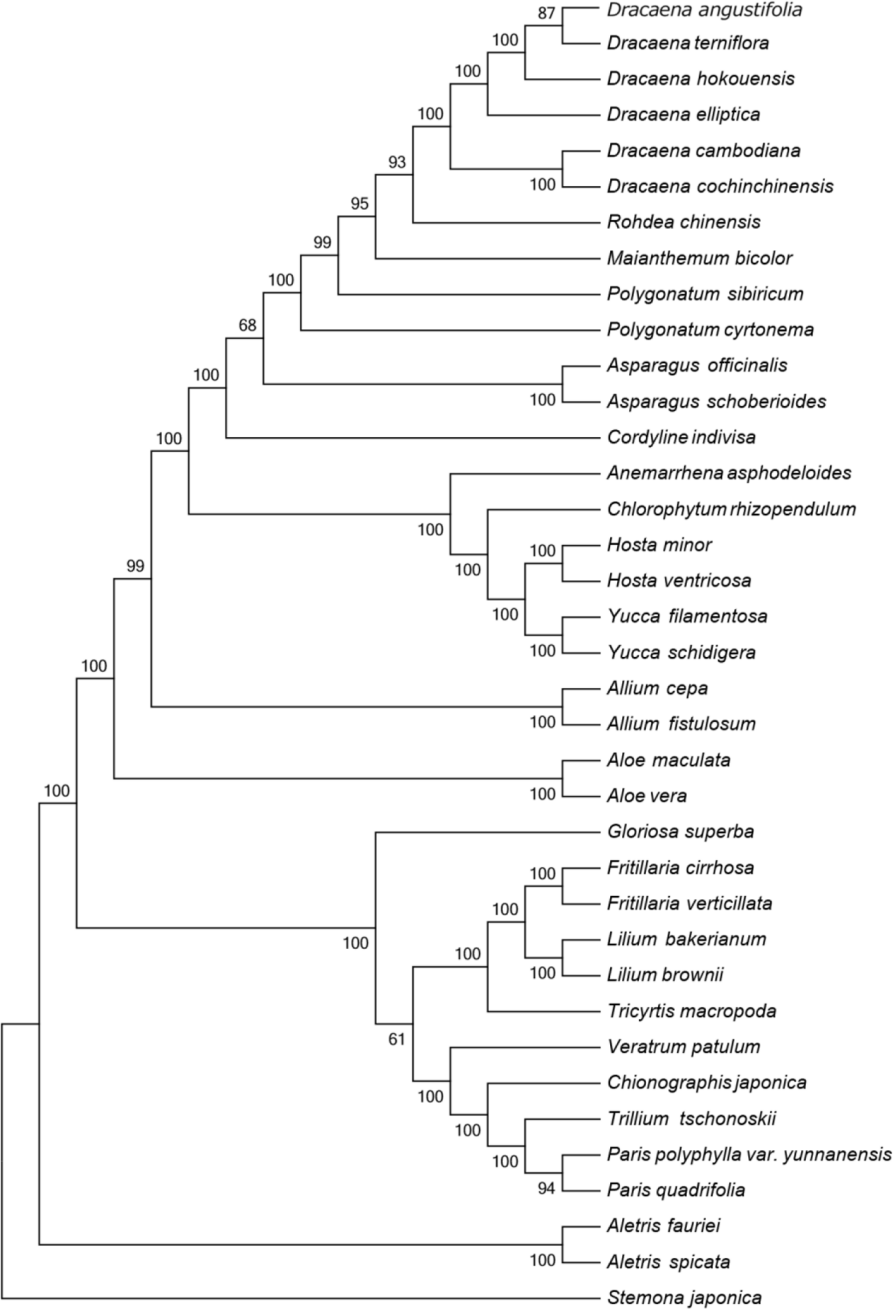
Phylogenetic tree constructed using MP based on complete CP genomes of six *Dracaena* and other 31 species. Numbers above the branches are the bootstrap support values.

The authors apologize for this error and state that this does not change the scientific conclusions of the article in any way. The original article has been updated.

