# Supplementary material for: Species Identification of Dracaena Using the Complete Chloroplast Genome as a Super-Barcode
Source: Front Pharmacol. 2019 Nov 29;10:1441. doi: 10.3389/fphar.2019.01441 (PMC6901964; doi:10.3389/fphar.2019.01441)
Supplement: Supplementary file 1 [file DataSheet_1.docx]

Supplementary Material

**Table S1.** Base composition in the chloroplast genomes of the six *Dracaena* species

| **Species** | **Regions** | | **Positions** | **T(U) (%)** | | **C (%)** | **A (%)** | **G (%)** | **Length (bp)** |
| --- | --- | --- | --- | --- | --- | --- | --- | --- | --- |
| *D. cochinchinensis* | Total |  | | 32 | | 19.1 | 30.8 | 18.4 | 155449 |
|  | IRa |  | | 29 | | 22.2 | 28.4 | 20.7 | 26525 |
|  | IRb |  | | 28 | | 20.7 | 28.6 | 22.2 | 26525 |
|  | LSC |  | | 33 | | 18.3 | 31.6 | 17.3 | 83907 |
|  | SSC |  | | 35 | | 16.3 | 34.1 | 14.8 | 18492 |
|  | CDS |  | | 31 | | 17.8 | 30.8 | 20.3 | 77187 |
|  |  | 1st position | | 24 | | 18.7 | 30.9 | 26.9 | 25729 |
|  |  | 2nd position | | 32 | | 20.5 | 29.2 | 18.0 | 25729 |
|  |  | 3rd position | | 38 | | 14.3 | 32.2 | 15.9 | 25729 |
| *D. cambodiana* | Total |  | | 32 | | 19.1 | 30.8 | 18.4 | 155291 |
|  | IRa |  | | 29 | | 22.2 | 28.4 | 20.7 | 26525 |
|  | IRb |  | | 28 | | 20.7 | 28.6 | 22.2 | 26525 |
|  | LSC |  | | 33 | | 18.3 | 31.6 | 17.3 | 83752 |
|  | SSC |  | | 35 | | 16.3 | 34.1 | 14.8 | 18489 |
|  | CDS |  | | 31 | | 17.8 | 30.7 | 20.3 | 77202 |
|  |  | 1st position | | 24 | | 18.7 | 30.9 | 26.9 | 25734 |
|  |  | 2nd position | | 32 | | 20.5 | 29.2 | 18.0 | 25734 |
|  |  | 3rd position | | 38 | | 14.3 | 32.2 | 15.9 | 25734 |
| *D.angustifolia* | Total |  | | 32 | | 19.1 | 30.9 | 18.4 | 155332 |
|  | IRa |  | | 29 | | 22.1 | 28.5 | 20.8 | 26530 |
|  | IRb |  | | 28 | | 20.8 | 28.6 | 22.1 | 26530 |
|  | LSC |  | | 33 | | 18.2 | 31.6 | 17.2 | 83807 |
|  | SSC |  | | 35 | | 16.4 | 34.1 | 14.8 | 18465 |
|  | CDS |  | | 31 | | 17.8 | 30.8 | 20.2 | 78732 |
|  |  | 1st position | | 24 | | 18.7 | 30.8 | 26.8 | 26244 |
|  |  | 2nd position | | 32 | | 20.4 | 29.3 | 17.9 | 26244 |
|  |  | 3rd position | | 38 | | 14.2 | 32.2 | 15.9 | 26244 |
| *D. terniflora* | Total |  | | 32 | | 19.1 | 30.8 | 18.4 | 155347 |
|  | IRa |  | | 29 | | 22.1 | 28.5 | 20.8 | 26530 |
|  | IRb |  | | 28 | | 20.8 | 28.6 | 22.1 | 26530 |
|  | LSC |  | | 33 | | 18.2 | 31.6 | 17.3 | 83794 |
|  | SSC |  | | 35 | | 16.4 | 34.0 | 14.8 | 18493 |
|  | CDS |  | | 31 | | 17.8 | 30.8 | 20.2 | 78744 |
|  |  | 1st position | | 24 | | 18.7 | 30.8 | 26.8 | 26248 |
|  |  | 2nd position | | 32 | | 20.4 | 29.3 | 17.8 | 26248 |
|  |  | 3rd position | | 38 | | 14.2 | 32.2 | 15.9 | 26248 |
| *D. hokouensis* | Total |  | | 32 | | 19.1 | 30.8 | 18.4 | 155340 |
|  | IRa |  | | 29 | | 22.1 | 28.5 | 20.8 | 26525 |
|  | IRb |  | | 28 | | 20.8 | 28.6 | 22.1 | 26525 |
|  | LSC |  | | 33 | | 18.2 | 31.6 | 17.3 | 83796 |
|  | SSC |  | | 35 | | 16.4 | 34.0 | 14.8 | 18494 |
|  | CDS |  | | 31 | | 17.8 | 30.8 | 20.2 | 78744 |
|  |  | 1st position | | 24 | | 18.8 | 30.8 | 26.7 | 26248 |
|  |  | 2nd position | | 32 | | 20.4 | 29.3 | 17.9 | 26248 |
|  |  | 3rd position | | 38 | | 14.3 | 32.2 | 15.9 | 26248 |
| *D. elliptica* | Total |  | | 32 | | 19.1 | 30.8 | 18.4 | 155055 |
|  | IRa |  | | 29 | | 22.1 | 28.5 | 20.8 | 26489 |
|  | IRb |  | | 28 | | 20.8 | 28.6 | 22.1 | 26489 |
|  | LSC |  | | 33 | | 18.2 | 31.6 | 17.3 | 83621 |
|  | SSC |  | | 35 | | 16.4 | 34.1 | 14.8 | 18456 |
|  | CDS |  | | 31 | | 17.8 | 30.8 | 20.2 | 77130 |
|  |  | 1st position | | 24 | | 18.7 | 30.9 | 26.9 | 25710 |
|  |  | 2nd position | | | 32 | 20.5 | 29.2 | 17.9 | 25710 |
|  |  | 3rd position | | | 38 | 14.3 | 32.2 | 15.9 | 25710 |

**Table S2.** The codon–anticodon recognition pattern and codon usage for the *Dracaena* chloroplast genome

| Amino acid | Codon | No.2 | No.3 | No.4 | No.5 | No.6 | No.7 | RSCU2 | RSCU3 | RSCU4 | RSCU5 | RSCU6 | RSCU7 | tRNA |
| --- | --- | --- | --- | --- | --- | --- | --- | --- | --- | --- | --- | --- | --- | --- |
| Phe | UUU | 894 | 892 | 930 | 930 | 926 | 895 | 1.24 | 1.24 | 1.25 | 1.25 | 1.25 | 1.24 |  |
| Phe | UUC | 547 | 550 | 559 | 559 | 557 | 550 | 0.76 | 0.76 | 0.75 | 0.75 | 0.75 | 0.76 | trnF-GAA |
| Leu | UUA | 822 | 822 | 845 | 844 | 844 | 816 | 1.87 | 1.87 | 1.88 | 1.88 | 1.87 | 1.87 | trnL-UAA |
| Leu | UUG | 533 | 534 | 547 | 547 | 547 | 532 | 1.21 | 1.22 | 1.22 | 1.22 | 1.22 | 1.22 | trnL-CAA |
| Leu | CUU | 559 | 556 | 576 | 576 | 577 | 557 | 1.27 | 1.27 | 1.28 | 1.28 | 1.28 | 1.28 |  |
| Leu | CUC | 189 | 189 | 189 | 190 | 192 | 189 | 0.43 | 0.43 | 0.42 | 0.42 | 0.43 | 0.43 |  |
| Leu | CUA | 367 | 366 | 374 | 374 | 374 | 363 | 0.84 | 0.83 | 0.83 | 0.83 | 0.83 | 0.83 | trnL-UAG |
| Leu | CUG | 165 | 166 | 167 | 167 | 167 | 162 | 0.38 | 0.38 | 0.37 | 0.37 | 0.37 | 0.37 |  |
| Ile | AUU | 1058 | 1057 | 1079 | 1079 | 1076 | 1059 | 1.43 | 1.43 | 1.42 | 1.42 | 1.42 | 1.42 |  |
| Ile | AUC | 465 | 464 | 475 | 473 | 473 | 468 | 0.63 | 0.63 | 0.63 | 0.62 | 0.62 | 0.63 | trnI-GAU |
| Ile | AUA | 703 | 704 | 725 | 725 | 723 | 710 | 0.95 | 0.95 | 0.95 | 0.96 | 0.95 | 0.95 | trnI-CAU |
| Met | AUG | 617 | 618 | 625 | 623 | 626 | 617 | 1 | 1 | 1 | 1 | 1 | 1 | trn(f)M-CAU |
| Val | GUU | 511 | 512 | 525 | 525 | 525 | 515 | 1.47 | 1.47 | 1.47 | 1.47 | 1.47 | 1.47 |  |
| Val | GUC | 169 | 170 | 174 | 174 | 176 | 171 | 0.48 | 0.49 | 0.49 | 0.49 | 0.49 | 0.49 | trnV-GAC |
| Val | GUA | 516 | 516 | 530 | 531 | 532 | 519 | 1.48 | 1.48 | 1.49 | 1.49 | 1.49 | 1.48 | trnV-UAC |
| Val | GUG | 198 | 198 | 198 | 199 | 197 | 195 | 0.57 | 0.57 | 0.56 | 0.56 | 0.55 | 0.56 |  |
| Ser | UCU | 563 | 564 | 575 | 578 | 574 | 559 | 1.69 | 1.69 | 1.68 | 1.69 | 1.68 | 1.68 |  |
| Ser | UCC | 340 | 339 | 345 | 342 | 345 | 338 | 1.02 | 1.01 | 1.01 | 1 | 1.01 | 1.02 | trnS-GGA |
| Ser | UCA | 420 | 421 | 435 | 433 | 432 | 421 | 1.26 | 1.26 | 1.27 | 1.27 | 1.27 | 1.26 | trnS-UGA |
| Ser | UCG | 182 | 182 | 190 | 189 | 190 | 183 | 0.54 | 0.54 | 0.56 | 0.55 | 0.56 | 0.55 |  |
| Pro | CCU | 398 | 400 | 404 | 406 | 405 | 398 | 1.49 | 1.49 | 1.49 | 1.49 | 1.49 | 1.49 |  |
| Pro | CCC | 232 | 229 | 227 | 230 | 232 | 227 | 0.87 | 0.85 | 0.84 | 0.85 | 0.85 | 0.85 |  |
| Pro | CCA | 311 | 313 | 318 | 315 | 316 | 314 | 1.16 | 1.17 | 1.17 | 1.16 | 1.16 | 1.17 | trnP-UGG |
| Pro | CCG | 131 | 131 | 136 | 136 | 136 | 132 | 0.49 | 0.49 | 0.5 | 0.5 | 0.5 | 0.49 |  |
| Thr | ACU | 525 | 526 | 529 | 532 | 532 | 529 | 1.59 | 1.6 | 1.6 | 1.6 | 1.6 | 1.61 |  |
| Thr | ACC | 234 | 236 | 238 | 238 | 240 | 235 | 0.71 | 0.72 | 0.72 | 0.72 | 0.72 | 0.71 | trnT-GGU |
| Thr | ACA | 407 | 406 | 407 | 407 | 406 | 403 | 1.24 | 1.23 | 1.23 | 1.22 | 1.22 | 1.22 | trnT-UGU |
| Thr | ACG | 152 | 151 | 152 | 152 | 152 | 149 | 0.46 | 0.46 | 0.46 | 0.46 | 0.46 | 0.45 |  |
| Ala | GCU | 619 | 621 | 625 | 623 | 620 | 616 | 1.79 | 1.79 | 1.79 | 1.79 | 1.78 | 1.78 |  |
| Ala | GCC | 218 | 217 | 215 | 215 | 217 | 217 | 0.63 | 0.63 | 0.62 | 0.62 | 0.62 | 0.63 |  |
| Ala | GCA | 410 | 413 | 408 | 405 | 407 | 407 | 1.18 | 1.19 | 1.17 | 1.16 | 1.17 | 1.18 | trnA-UGC |
| Ala | GCG | 140 | 137 | 145 | 149 | 147 | 145 | 0.4 | 0.39 | 0.42 | 0.43 | 0.42 | 0.42 |  |
| Tyr | UAU | 726 | 726 | 757 | 755 | 759 | 726 | 1.58 | 1.58 | 1.59 | 1.59 | 1.59 | 1.58 |  |
| Tyr | UAC | 192 | 192 | 197 | 197 | 195 | 191 | 0.42 | 0.42 | 0.41 | 0.41 | 0.41 | 0.42 | trnY-GUA |
| Stop | UAA | 42 | 41 | 41 | 41 | 41 | 41 | 1.5 | 1.46 | 1.45 | 1.45 | 1.45 | 1.46 |  |
| Stop | UAG | 22 | 22 | 22 | 22 | 23 | 22 | 0.79 | 0.79 | 0.78 | 0.78 | 0.81 | 0.79 |  |
| His | CAU | 497 | 496 | 521 | 520 | 519 | 497 | 1.53 | 1.53 | 1.55 | 1.55 | 1.54 | 1.54 |  |
| His | CAC | 151 | 151 | 151 | 152 | 153 | 150 | 0.47 | 0.47 | 0.45 | 0.45 | 0.46 | 0.46 | trnH-GUG |
| Gln | CAA | 673 | 674 | 691 | 694 | 692 | 674 | 1.51 | 1.5 | 1.51 | 1.51 | 1.5 | 1.5 | trnQ-UUG |
| Gln | CAG | 221 | 222 | 227 | 227 | 228 | 222 | 0.49 | 0.5 | 0.49 | 0.49 | 0.5 | 0.5 |  |
| Asn | AAU | 936 | 938 | 954 | 959 | 957 | 937 | 1.51 | 1.51 | 1.51 | 1.51 | 1.51 | 1.51 |  |
| Asn | AAC | 305 | 304 | 309 | 309 | 311 | 306 | 0.49 | 0.49 | 0.49 | 0.49 | 0.49 | 0.49 | trnN-GUU |
| Lys | AAA | 1015 | 1012 | 1036 | 1037 | 1038 | 1014 | 1.48 | 1.48 | 1.48 | 1.48 | 1.48 | 1.48 | trnK-UUU |
| Lys | AAG | 354 | 356 | 362 | 362 | 360 | 355 | 0.52 | 0.52 | 0.52 | 0.52 | 0.52 | 0.52 |  |
| Asp | GAU | 834 | 836 | 851 | 850 | 850 | 833 | 1.59 | 1.6 | 1.6 | 1.59 | 1.59 | 1.59 |  |
| Asp | GAC | 214 | 212 | 215 | 216 | 217 | 213 | 0.41 | 0.4 | 0.4 | 0.41 | 0.41 | 0.41 | trnD-GUC |
| Glu | GAA | 982 | 984 | 1012 | 1011 | 1009 | 982 | 1.48 | 1.48 | 1.49 | 1.49 | 1.49 | 1.48 | trnE-UUC |
| Glu | GAG | 348 | 348 | 346 | 347 | 347 | 344 | 0.52 | 0.52 | 0.51 | 0.51 | 0.51 | 0.52 |  |
| Cys | UGU | 233 | 234 | 239 | 237 | 238 | 233 | 1.54 | 1.54 | 1.54 | 1.54 | 1.53 | 1.54 |  |
| Cys | UGC | 70 | 69 | 71 | 71 | 73 | 69 | 0.46 | 0.46 | 0.46 | 0.46 | 0.47 | 0.46 | trnC-GCA |
| Stop | UGA | 20 | 21 | 22 | 22 | 21 | 21 | 0.71 | 0.75 | 0.78 | 0.78 | 0.74 | 0.75 |  |
| Trp | UGG | 453 | 453 | 462 | 462 | 462 | 453 | 1 | 1 | 1 | 1 | 1 | 1 | trnW-CCA |
| Arg | CGU | 351 | 351 | 362 | 361 | 362 | 355 | 1.33 | 1.33 | 1.35 | 1.35 | 1.35 | 1.35 | trnR-ACG |
| Arg | CGC | 87 | 87 | 84 | 86 | 85 | 85 | 0.33 | 0.33 | 0.31 | 0.32 | 0.32 | 0.32 |  |
| Arg | CGA | 349 | 349 | 361 | 358 | 362 | 350 | 1.32 | 1.32 | 1.35 | 1.34 | 1.35 | 1.33 |  |
| Arg | CGG | 125 | 124 | 126 | 128 | 125 | 124 | 0.47 | 0.47 | 0.47 | 0.48 | 0.46 | 0.47 |  |
| Arg | AGA | 396 | 397 | 401 | 401 | 401 | 393 | 1.19 | 1.19 | 1.17 | 1.18 | 1.17 | 1.18 | trnR-UCU |
| Arg | AGG | 103 | 103 | 104 | 104 | 106 | 103 | 0.31 | 0.31 | 0.3 | 0.3 | 0.31 | 0.31 |  |
| Ser | AGU | 515 | 512 | 515 | 516 | 519 | 509 | 1.95 | 1.94 | 1.92 | 1.93 | 1.93 | 1.93 |  |
| Ser | AGC | 159 | 158 | 160 | 159 | 160 | 156 | 0.6 | 0.6 | 0.6 | 0.59 | 0.6 | 0.59 | trnS-GCU |
| Gly | GGU | 567 | 565 | 574 | 573 | 569 | 569 | 1.29 | 1.28 | 1.29 | 1.29 | 1.28 | 1.29 |  |
| Gly | GGC | 172 | 173 | 171 | 173 | 174 | 171 | 0.39 | 0.39 | 0.38 | 0.39 | 0.39 | 0.39 | trnG-GCC |
| Gly | GGA | 729 | 728 | 729 | 728 | 727 | 724 | 1.66 | 1.65 | 1.64 | 1.64 | 1.64 | 1.64 | trnG-UCC |
| Gly | GGG | 293 | 296 | 304 | 304 | 302 | 297 | 0.67 | 0.67 | 0.68 | 0.68 | 0.68 | 0.67 |  |

**Table S3** Gene information in the six CP genomes of *Dracaena* species

| **Regions Species** | *D. cochinchinensis* | | | |
| --- | --- | --- | --- | --- |
|  | No. | Duplication | Length/bp | % |
| Protein-coding Genes (CDS) | 84 | 7 | 77187 | 49.6542274 |
| rRNAs | 8 | 4 | 9050 | 5.82184511 |
| tRNAs | 38 | 8 | 2874 | 1.84883788 |
| **Regions Species** | *D. cambodiana* | | | |
|  | No. |  | Length/bp | % |
| Protein-coding Genes (CDS) | 84 | 7 | 77202 | 49.7144071 |
| rRNAs | 8 | 4 | 9050 | 5.82776851 |
| tRNAs | 38 | 8 | 2874 | 1.85071897 |
| **Regions Species** | *D. angustifolia* | | | |
|  | No. |  | Length/bp | % |
| Protein-coding Genes (CDS) | 85 | 7 | 78732 | 50.686272 |
| rRNAs | 8 | 4 | 9050 | 5.82623027 |
| tRNAs | 38 | 8 | 2873 | 1.84958669 |
| **Regions Species** | *D. terniflora* | | | |
|  | No. |  | Length/bp | % |
| Protein-coding Genes (CDS) | 85 | 7 | 78744 | 50.6891025 |
| rRNAs | 8 | 4 | 9050 | 5.8256677 |
| tRNAs | 38 | 8 | 2873 | 1.8494081 |
| **Regions Species** | *D. hokouensis* | | | |
|  | No. |  | Length/bp | % |
| Protein-coding Genes (CDS) | 85 | 7 | 78744 | 50.6913866 |
| rRNAs | 8 | 4 | 9050 | 5.82593022 |
| tRNAs | 38 | 8 | 2874 | 1.85013519 |
| **Regions Species** | *D. elliptica* | | | |
|  | No. |  | Length/bp | % |
| Protein-coding Genes (CDS) | 84 | 7 | 77130 | 49.7436394 |
| rRNAs | 8 | 4 | 9050 | 5.83663861 |
| tRNAs | 38 | 8 | 2874 | 1.85353584 |

**Table S4** Genes with introns in the chloroplast genomes of the six *Dracaena* species as well as the lengths of the exons and introns

| *D.cochinchinensis* | | | | | | |
| --- | --- | --- | --- | --- | --- | --- |
| gene | length(bp) | | | | | |
|  | location | Exon1(bp) | Intron1(bp) | Exon2(bp) | Intron2(bp) | Exon3(bp) |
| trnK-UUU |  | 12 | 859 | 11 |  |  |
| rps16 |  | 15 | 291 | 69 |  |  |
| trnG-UCC |  | 7 | 234 | 15 |  |  |
| atpF |  | 47 | 280 | 134 |  |  |
| rpoC1 |  | 143 | 236 | 545 |  |  |
| ycf3 |  | 41 | 249 | 76 | 245 | 50 |
| trnL-UAA |  | 11 | 179 | 16 |  |  |
| trnV-UAC |  | 12 | 195 | 12 |  |  |
| rps12 |  | 38 |  |  |  |  |
| clpP |  | 23 | 273 | 97 | 216 | 75 |
| petB |  | 2 | 250 | 213 |  |  |
| petD |  | 2 | 248 | 168 |  |  |
| rpl16 |  | 2 | 335 | 132 |  |  |
| rpl2 |  | 130 | 217 | 143 |  |  |
| ndhB |  | 258 | 232 | 251 |  |  |
| trnl-GAU |  | 13 | 312 | 11 |  |  |
| trnA-UGC |  | 12 | 271 | 11 |  |  |
| ndhA |  | 185 | 359 | 179 |  |  |
| rps12 |  |  | 180 | 77 |  | 8 |
| trnA-UGC |  | 12 | 271 | 11 |  |  |
| trnl-GAU |  | 13 | 311 | 11 |  |  |
| rps12 |  |  | 180 | 77 |  | 8 |
| ndhB |  | 258 | 232 | 251 |  |  |
| rpl2 |  | 130 | 216 | 143 |  |  |
| *D.cambodiana* | | | | | | |
| gene | length(bp) | | | | | |
|  | location | Exon1(bp) | Intron1(bp) | Exon2(bp) | Intron2(bp) | Exon3(bp) |
| trnK-UUU |  | 12 | 857 | 11 |  |  |
| rps16 |  | 15 | 291 | 69 |  |  |
| trnG-UCC |  | 7 | 235 | 15 |  |  |
| atpF |  | 48 | 280 | 134 |  |  |
| rpoC1 |  | 143 | 236 | 545 |  |  |
| ycf3 |  | 41 | 242 | 76 | 247 | 50 |
| trnL-UAA |  | 11 | 179 | 16 |  |  |
| trnV-UAC |  | 13 | 195 | 11 |  |  |
| rps12 |  | 37 |  |  |  |  |
| clpP |  | 22 | 272 | 96 | 214 | 75 |
| petB |  | 1 | 250 | 213 |  |  |
| petD |  | 2 | 248 | 167 |  |  |
| rpl16 |  | 2 | 338 | 133 |  |  |
| rpl2 |  | 129 | 217 | 143 |  |  |
| ndhB |  | 259 | 233 | 252 |  |  |
| rps12 |  |  | 180 | 76 |  | 8 |
| trnl-GAU |  | 14 | 312 | 11 |  |  |
| trnA-UGC |  | 12 | 272 | 11 |  |  |
| ndhA |  | 186 | 357 | 179 |  |  |
| trnA-UGC |  | 12 | 272 | 11 |  |  |
| trnl-GAU |  | 14 | 312 | 11 |  |  |
| rps12 |  |  | 180 | 76 |  | 8 |
| ndhB |  | 259 | 233 | 252 |  |  |
| rpl2 |  | 129 | 217 | 143 |  |  |
| *D.angustifolia* | | | | | | |
| gene | length(bp) | | | | | |
|  | location | Exon1(bp) | Intron1(bp) | Exon2(bp) | Intron2(bp) | Exon3(bp) |
| trnK-UUU |  | 11 | 857 | 11 |  |  |
| rps16 |  | 15 | 289 | 69 |  |  |
| trnG-UCC |  | 7 | 235 | 16 |  |  |
| atpF |  | 48 | 275 | 134 |  |  |
| rpoC1 |  | 144 | 236 | 544 |  |  |
| ycf3 |  | 41 | 243 | 76 | 245 | 50 |
| trnL-UAA |  | 11 | 184 | 16 |  |  |
| trnV-UAC |  | 12 | 196 | 12 |  |  |
| rps12 |  | 38 |  |  |  |  |
| clpP |  | 22 | 273 | 97 | 205 | 74 |
| petB |  | 2 | 250 | 214 |  |  |
| petD |  | 2 | 248 | 168 |  |  |
| rpl16 |  | 2 | 344 | 132 |  |  |
| rpl2 |  | 130 | 216 | 143 |  |  |
| ndhB |  | 259 | 233 | 252 |  |  |
| rps12 |  |  | 180 | 76 |  | 8 |
| trnl-GAU |  | 14 | 314 | 11 |  |  |
| trnA-UGC |  | 12 | 271 | 11 |  |  |
| ndhA |  | 186 | 362 | 179 |  |  |
| trnA-UGC |  | 12 | 271 | 2 |  |  |
| trnl-GAU |  | 14 | 314 | 11 |  |  |
| rps12 |  |  | 180 | 76 |  | 8 |
| ndhB |  | 259 | 233 | 252 |  |  |
| rpl2 |  | 130 | 216 | 143 |  |  |
| *D.terniflora* | | | | | | |
| gene | length(bp) | | | | | |
|  | location | Exon1(bp) | Intron1(bp) | Exon2(bp) | Intron2(bp) | Exon3(bp) |
| trnK-UUU |  | 12 | 857 | 11 |  |  |
| rps16 |  | 15 | 289 | 69 |  |  |
| trnG-UCC |  | 7 | 235 | 16 |  |  |
| atpF |  | 48 | 281 | 134 |  |  |
| rpoC1 |  | 144 | 236 | 545 |  |  |
| ycf3 |  | 41 | 243 | 75 | 246 | 51 |
| trnL-UAA |  | 11 | 180 | 16 |  |  |
| trnV-UAC |  | 12 | 197 | 12 |  |  |
| rps12 |  | 37 |  |  |  |  |
| clpP |  | 22 | 272 | 96 | 205 | 74 |
| petB |  | 2 | 250 | 214 |  |  |
| petD |  | 2 | 248 | 168 |  |  |
| rpl16 |  | 3 | 348 | 133 |  |  |
| rpl2 |  | 129 | 217 | 143 |  |  |
| ndhB |  | 258 | 232 | 251 |  |  |
| rps12 |  |  | 180 | 77 |  | 8 |
| trnl-GAU |  | 13 | 313 | 11 |  |  |
| trnA-UGC |  | 12 | 271 | 11 |  |  |
| ndhA |  | 186 | 360 | 179 |  |  |
| trnA-UGC |  | 12 | 271 | 11 |  |  |
| trnl-GAU |  | 14 | 314 | 11 |  |  |
| rps12 |  |  | 180 | 76 |  | 8 |
| ndhB |  | 259 | 233 | 252 |  |  |
| rpl2 |  | 130 | 216 | 143 |  |  |
| *D.hokouensis* | | | | | | |
| gene | length(bp) | | | | | |
|  | location | Exon1(bp) | Intron1(bp) | Exon2(bp) | Intron2(bp) | Exon3(bp) |
| trnK-UUU |  | 12 | 857 | 11 |  |  |
| rps16 |  | 14 | 302 | 69 |  |  |
| trnG-UCC |  | 7 | 234 | 15 |  |  |
| atpF |  | 48 | 275 | 134 |  |  |
| rpoC1 |  | 144 | 237 | 544 |  |  |
| ycf3 |  | 41 | 243 | 76 | 246 | 50 |
| trnL-UAA |  | 11 | 181 | 16 |  |  |
| trnV-UAC |  | 13 | 197 | 11 |  |  |
| rps12 |  | 37 |  |  |  |  |
| clpP |  | 22 | 273 | 97 | 205 | 75 |
| petB |  | 1 | 249 | 213 |  |  |
| petD |  | 2 | 248 | 168 |  |  |
| rpl16 |  | 2 | 338 | 132 |  |  |
| rpl2 |  | 130 | 217 | 143 |  |  |
| ndhB |  | 258 | 232 | 251 |  |  |
| rps12 |  |  | 180 | 77 |  | 8 |
| trnl-GAU |  | 13 | 311 | 11 |  |  |
| trnA-UGC |  | 12 | 271 | 11 |  |  |
| ndhA |  | 179 | 366 | 186 |  |  |
| trnA-UGC |  | 12 | 271 | 11 |  |  |
| trnl-GAU |  | 13 | 311 | 11 |  |  |
| rps12 |  |  | 180 | 77 |  | 8 |
| ndhB |  | 258 | 232 | 251 |  |  |
| rpl2 |  | 130 | 217 | 143 |  |  |
| *D.elliptica* | | | | | | |
| gene | length(bp) | | | | | |
|  | location | Exon1(bp) | Intron1(bp) | Exon2(bp) | Intron2(bp) | Exon3(bp) |
| trnK-UUU |  | 12 | 856 | 11 |  |  |
| rps16 |  | 15 | 288 | 69 |  |  |
| trnG-UCC |  | 7 | 235 | 15 |  |  |
| atpF |  | 48 | 275 | 134 |  |  |
| rpoC1 |  | 144 | 236 | 544 |  |  |
| ycf3 |  | 41 | 240 | 76 | 247 | 51 |
| trnL-UAA |  | 11 | 182 | 16 |  |  |
| trnV-UAC |  | 12 | 194 | 12 |  |  |
| rps12 |  | 37 |  |  |  |  |
| clpP |  | 22 | 266 | 96 | 214 | 74 |
| petB |  | 1 | 253 | 213 |  |  |
| petD |  | 2 | 248 | 168 |  |  |
| rpl16 |  | 2 | 344 | 132 |  |  |
| rpl2 |  | 130 | 216 | 143 |  |  |
| ndhB |  | 259 | 233 | 252 |  |  |
| rps12 |  |  | 180 | 76 |  | 8 |
| trnl-GAU |  | 14 | 312 | 11 |  |  |
| trnA-UGC |  | 12 | 272 | 11 |  |  |
| ndhA |  | 179 | 358 | 186 |  |  |
| trnA-UGC |  | 12 | 271 | 11 |  |  |
| trnl-GAU |  | 13 | 311 | 11 |  |  |
| rps12 |  |  | 180 | 77 |  | 8 |
| ndhB |  | 258 | 232 | 251 |  |  |
| rpl2 |  | 130 | 217 | 143 |  |  |

**Table S5** GenBank information of complete chloroplast genome used for phylogenetic analyses in this study

| GenBank acc. | Species | genus |
| --- | --- | --- |
| NC_033412.1 | *Aletris fauriei* | *Aletris* |
| NC_033411.1 | *Aletris spicata* |  |
| NC_024813.1 | *Allium cepa* | *Allium* |
| NC_040222.1 | *Allium fistulosum* |  |
| NC_035505.1 | *Aloe maculata* | *Aloe* |
| NC_035506.1 | *Aloe vera* |  |
| NC_032698.1 | *Anemarrhena asphodeloides* | *Anemarrhena* |
| NC_035999.1 | *Hosta minor* | *Hosta* |
| NC_032706.1 | *Hosta ventricosa* |  |
| NC_032712.1 | *Yucca filamentosa* | *Yucca* |
| NC_032714.1 | *Yucca schidigera* |  |
| KX931454.1 | *Chlorophytum rhizopendulum* | *Chlorophytum* |
| NC_034777.1 | *Asparagus officinalis* | *Asparagus* |
| NC_035969.1 | *Asparagus schoberioides* |  |
| NC_035970.1 | *Maianthemum bicolor* | *Maianthemum* |
| MH356725.1 | *Rohdea chinensis* | *Rohdea* |
| NC_029485.1 | *Polygonatum sibiricum* | *Polygonatum* |
| NC_028429.1 | *Polygonatum cyrtonema* |  |
| NC_035998.1 | *Cordyline indivisa* | *Cordyline* |
| NC_024728.1 | *Fritillaria cirrhosa* | *Fritillaria* |
| NC_037217.1 | *Fritillaria verticillata* |  |
| NC_035592.1 | *Lilium bakerianum* | *Lilium* |
| NC_035588.1 | *Lilium brownii* |  |
| NC_040223.1 | *Tricyrtis macropoda* | *Tricyrtis* |
| KF951065.1 | *Chionographis japonica* | *Chionographis* |
| KT805945.1 | *Paris polyphylla* var. *yunnanensis* | *Paris* |
| NC_033520.1 | *Paris quadrifolia* |  |
| MF614015.1 | *Trillium tschonoskii* | *Trillium* |
| NC_022715.2 | *Veratrum patulum* | *Veratrum* |
| NC_030065.1 | *Gloriosa superba* | *Gloriosa* |
| NC_039675.1 | *Stemona japonica* | *Stemona* |


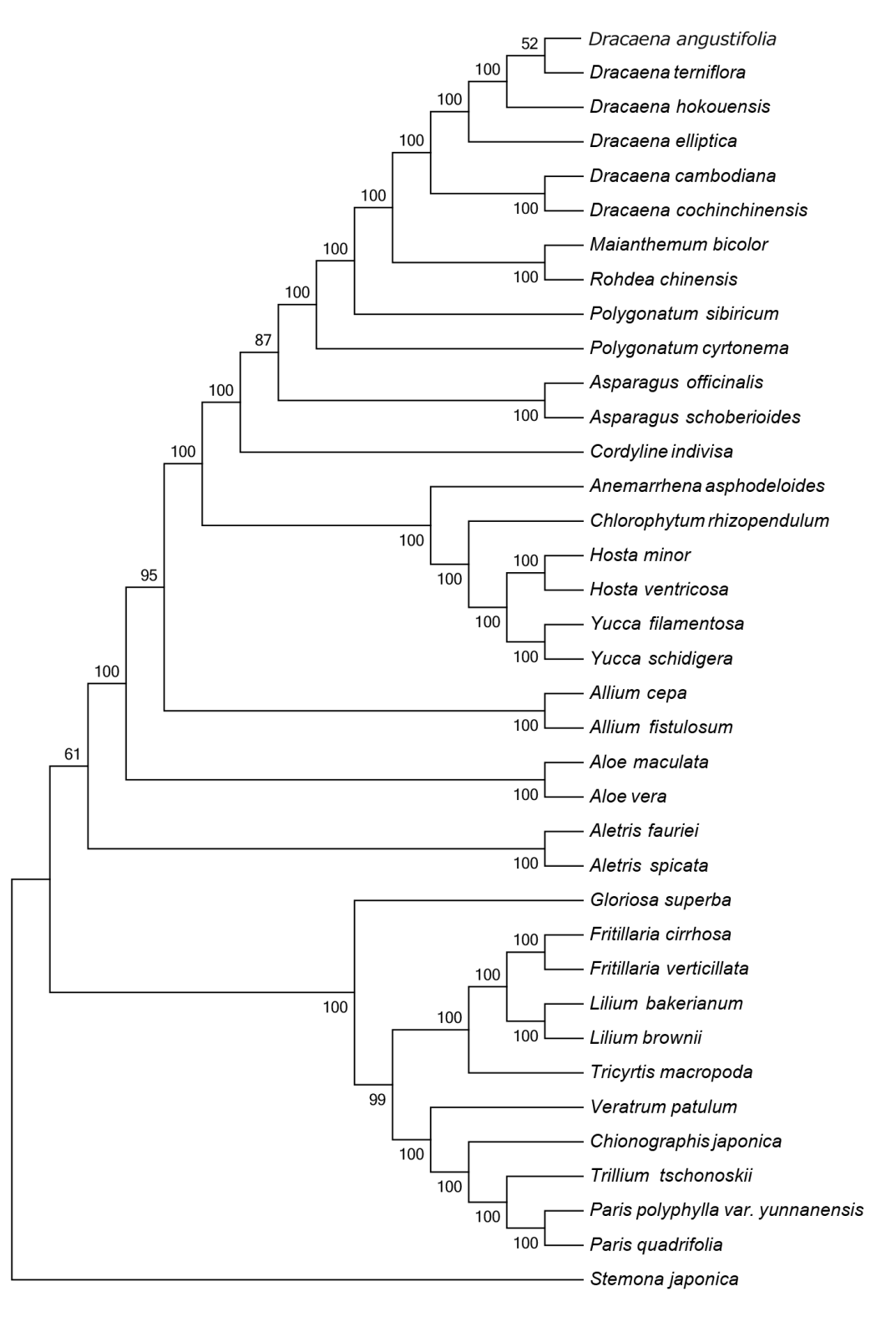


**Figure S1** Phylogenetic tree constructed using NJ based on complete CP genomes of six *Dracaena* and other 31 species. Numbers above the branches are bootstrap support values.
